# Adhesion Performance and Recovery of Acrylic PSA with Acrylic Elastomer (AE) Blends via Thermal Crosslinking for Application in Flexible Displays

**DOI:** 10.3390/polym11121959

**Published:** 2019-11-28

**Authors:** Jung-Hun Lee, Gyu-Seong Shim, Hyun-Joong Kim, Youngdo Kim

**Affiliations:** 1 Lab. of Adhesion and Bio-Composites; Program in Environmental Materials Science; Seoul National University, Seoul 08826, Korea; fujiisletter@naver.com (J.-H.L.); sks6567@snu.ac.kr (G.-S.S.); 2 Research Institute of Agriculture and Life Sciences; College of Agriculture and Life Sciences; Seoul National University, Seoul 08826, Korea; 3 Samsung Display Co., Ltd., Yongin 08826, Korea; colour.kim@samsung.com

**Keywords:** acrylic pressure-sensitive adhesives, acrylic elastomer, thermal crosslinking, adhesion performance, recovery

## Abstract

Acrylic pressure-sensitive adhesive (PSA) is used to fix each layer of a flexible display. Acrylic PSA needs to satisfy specific elongation and recovery requirements so that reliability of the flexible display can be achieved. For this reason, we aimed to design an acrylic PSA/acrylic elastomer (AE) blend and to study how some viscoelastic and adhesion properties are influenced by the AE content into the mixed, blended system. Samples were characterized by UV–Vis spectrophotometry for transmittance, texture analysis for adhesion performances, and dynamic mechanical analysis (DMA) for recovery and viscoelasticity. When acrylic PSA/AE was simply blended, the adhesion performance changed due to the influence of the long molecular chains of AE. Based on this result, the AE content was fixed at 10 wt %, and acrylic PSA prepolymer was crosslinked at different concentrations of crosslinking agent. Peel strength and probe tack decreased as the concentration of crosslinking agent increased, as reported in previous studies. On the other hand, as the content of the crosslinking agent increased, recovery characteristics were improved. Additionally, as the content of the crosslinking agent increased, the storage modulus also increased, although the glass-transition temperature was not affected. According to these findings, we successfully proved the possibility of using AE to adjust adhesion performance and recovery of acrylic PSA for designing flexible displays.

## 1. Introduction

Pressure-sensitive adhesives (PSAs) are semisolid phase materials used to bond the surfaces of various materials primarily by adhesion and cohesion. Applying a light contact pressure for a short time shows good viscoelasticity and adhesion performance to the solid substrate. In general, PSA needs to exhibit various physical properties depending on the characteristics of the product to which it is applied; these products range from a variety of industries, including the medical, electronics, construction, and automobile industries [1]. In particular, acrylic PSAs have many advantages, such as excellent aging characteristics, high temperature resistance, and excellent optical transparency [2]. In addition, acrylic PSAs have the advantage that their properties can be controlled by molecular weight, structure, and functional groups [3]. Recently, interest in developing flexible displays has rapidly increased, and improving the functionality of acrylic PSAs is an emerging necessity. This is because an acrylic PSA strongly related to this task must withstand the stress and strain that occur when the display folds or bends.

Elastomer is a polymer having viscoelasticity, weak intermolecular attraction, generally low Young’s modulus, and failure strain higher than that of other materials [4]. Since the elastomer maintains the amorphous polymer form above its glass-transition temperature, molecular reconformation is possible without destruction of covalent bonds. Among the various elastomers, acrylic elastomer (AE), also known as acrylic rubber (ACM), is a copolymer of methyl or ethyl acrylate. To improve oil resistance, processability, and damping ability, the mixing of ACM with a thermoplastic polymer has been reported in a number of studies, where the thermoplastic components involved were poly(lactic acid) [5], nylon [6,7,8], poly(vinylidene fluoride) [9,10], poly(vinyl chloride) [11], poly(ethylene terephtalate) [12], or chlorinated polypropylene [13]. 

In our previous research, we conducted studies to find adhesion performance and recovery of an acrylic PSA according to the degree of crosslinking as a highly important factor in controlling the adhesive behavior. However, there was a limit to applying an acrylic PSA to a flexible display by only controlling the degree of crosslinking. This is because when this quantity increases beyond a certain level, problems arise that cannot tolerate specific deformations [14]. We successfully obtained valuable physical properties (adhesion and recovery) for crosslinked blends relayed onto acrylic PSA/styrene-isoprene elastomer. In this case, although the effect of the elastomer with a long molecular chain certainly influenced the recovery, there was a limitation in that the acrylic PSA became opaque and difficult to apply as a display material [15].

**Figure 1:**
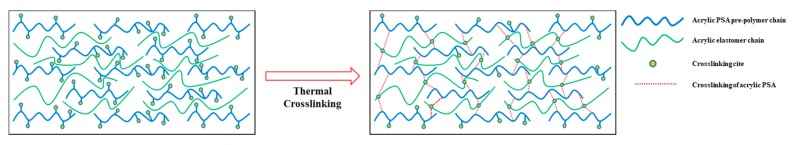
Schematic design of acrylic pressure-sensitive adhesive (PSA) and acrylic elastomer (AE) via the crosslinking reaction.

In this research, acrylic PSA and AE were simply blended to confirm adhesion performance and recovery of the mixed system. Based on this result, the optimum AE content was fixed. Acrylic PSA was crosslinked using different concentrations of the diisocyanate (as crosslinker) in an attempt to grasp the acrylic elastomer chains through the crosslinkages established between the PSA molecules (see Figure 1). Peel strength, probe tack, and lap shear tests were performed using a sticky texture analyzer, and recovery characteristics were analyzed by a stress relaxation test using dynamic mechanical analysis (DMA).

## 2. Experimental Section

### 2.1. Materials

Acrylic PSA prepolymer (Cosmotec Co., Ltd., Tokyo, Japan) and acrylic elastomer (AE, Tohpe Corp., Tokyo, Japan) were used as received without further purification. Ethyl acetate (EAc, 99.0% purity, Samchun Pure Chemical, Seoul, Republic of Korea) was used as a solvent. An AK-75 diisocyanate compound (Aekyung Chemical Corp. Ltd., Seoul, Republic of Korea) with an NCO content between 12.5% and 13.5% was used as a crosslinking agent for acrylic PSA prepolymer chains.

### 2.2. Non-Crosslinked Acrylic PSA/AE Blends

The acrylic PSA prepolymer was mixed with AE to find out the optimum AE content for use as a PSA sheet. These samples were spread on polyethylene terephthalate (PET) films without thermal crosslinking to investigate the influence of AE on adhesion properties and recovery of acrylic PSA and then dried at a temperature of 80 °C for 10 min to evaporate the EAc.

### 2.3. Thermal Crosslinked Acrylic PSA/AE Blends

Crosslinked acrylic PSA with AE was prepared by blending acrylic PSA/AE (90 wt %/10 wt %) with 0.2, 0.4, 0.6, 0.8, or 1.0 phr of the crosslinking agent. Based on the investigations carried out on the non-crosslinked PSA/AE blends, AE content was eventually chosen to be 10 wt %. Such mixed samples with different contents of crosslinker were deposited onto the surface of 75 μm thick corona-treated PET films and then dried at a temperature of 80 °C for 10 min, followed by thermal crosslinking at 120 °C for 2 min and at room temperature for 24 h.

### 2.4. Characterizations

Molecular weights and polydispersity indices for the acrylic PSA and AE were measured using gel permeation chromatography (GPC), an Agilent 1100 instrument (Agilent Technologies, Inc., Santa Clara, CA, USA), equipped with a pump and a refractive index detector. Tetrahydrofuran was used as an eluent, and the flow rate was 1 mL/min.

Glass-transition temperatures for the synthesized acrylic PSAs were determined by differential scanning calorimetry (DSC; Q-200, TA Instrument, New Castel, DE, USA). The samples were first scanned from room temperature to 150 °C at a heating rate of 5 °C/min, after which they were rapidly cooled to −80 °C and then scanned again up to 0 °C at the same heating rate. During the analysis, glass-transition temperatures of the acrylic PSAs were measured between −80 °C and 0 °C after annealing to higher temperatures in order to exclude possible thermal history effects.

Visible light transmittances of the specimens were measured at wavelengths ranging from 400 to 800 nm using a UV–Vis spectrophotometer (Cary 100, Agilent Technologies, Santa Clara, CA, USA). The thicknesses of all acrylic PSA films deposited on transparent PET substrates were 75 μm.

The peel strengths of specimens with widths of 25 mm were investigated using a texture analyzer (TA-T2i, Micro Stable Systems, London, UK). The specimens were pressed onto stainless steel (SUS) substrates by two passes of a 2 kg rubber roller and then aged at room temperature for 24 h. The peel strength magnitude (defined as the average force applied to a PSA specimen during debonding) was determined in accordance with the ASTM D3330 standard at an angle of 180°, crosshead rate of 300 mm/min, and temperature of 20 °C. The applied force was recorded for six different trials with an average value of N/25 mm.

The probe tack procedure was conducted at a temperature of 20 °C in accordance with the ASTM D2979 standard using a texture analyzer equipped with a 5 mm diameter SUS cylinder probe. The standard probe tack testing procedure was utilized, consisting of the following three stages, namely, approaching the PSA surface, touching the PSA surface, and detaching from the PSA surface. The probe rate was 0.5 mm/s, the separation rate was 10 mm/s, and the contact time with the PSA surface was 1 s at a constant force of 100 gf/cm^2^. During debonding, the measured tack values corresponded to the maximum debonding forces. The debonding forces were recorded six times with an average value of N.

Lap shear testing was investigated using a texture analyzer. The tested specimens were cut into smaller pieces 25 mm in width. After being removed from a silicone release film, each PSA film was attached to another PET substrate (the adhesion cross-sectional area was equal to 25 × 25 mm^2^, and a 2 kg rubber roller was passed over the film surface three times). Lap shear tests were performed at a crosshead rate of 5 mm/min. Shear strain rate values were calculated using the following equation:

Shear strain rate (%) = *ΔL/t* × 100, (1)

where *ΔL* is the moving distance and *t* is the thickness of the PSA film.

PSAs for flexible displays are usually subjected to different shear strain values depending on their structure and radius of curvature. Therefore, researching the relationship between the shear strain rate and thickness of the applied PSA films is imperative for supporting the next use of PSAs in the flexible display industry [16].

Stress relaxation testing was conducted using a DMA apparatus (Q-800 TA Instruments, New Castel, DE, USA). The goal of this experiment was to determine PSA characteristics and their suitability for application in flexible displays by assessing the correlation between deformation and stress over time. During the initial testing period, the PSA samples being investigated were stabilized for 1 min, and then 400% strain was applied for 10 min. Afterwards, specimens were left to rest for 5 min. Degrees of elastic recovery and residual creep strain values were measured for the PSA samples as a function of time at different applied strain magnitudes. Initial stress, final stress, and relaxation ratio values were determined from the obtained stress/time graphs [16].

Viscoelasticity, storage modulus, and tan δ of the acrylic PSA/AE blends as a function of the crosslinking agent content were measured using a DMA analyzer in the shear sandwich mode. Specimens of approximately 5 mm in length, 5 mm in width, and 0.5 mm in thickness were prepared. DMA was performed using a multi-strain mode with a heating rate of 3 °C/min over a temperature range between −80 °C and 100 °C.

## 3. Results and Discussion

### 3.1. Basic Properties of Acrylic PSA and AE

Table 1 shows the molecular weights (M_n_ and M_w_) and polydispersity indices (PDIs) for acrylic PSA and AE. The number average molecular weights of acrylic PSA and AE were approximately 25,000 g/mol and 340,000 g/mol, respectively. The PDIs of the acrylic PSA and AE were 2.1 and 3.4, respectively. Despite a certain nonuniformity expressed by the two different PDI values, the prepolymer and elastomer components displayed a good mutual compatibility during the mixing processes.

Table 1 also shows the values of glass-transition temperature (*T_g_*) for acrylic PSA and AE. The T_g_ is an important factor, which influences some physical properties of PSA film. The *T_g_* may affect the wettability of the PSA to the substrate and the modulus of elasticity of the adhesive itself. Based on the DSC data, the *T_g_* of the acrylic PSA was approximately 10 °C lower than that of AE due to AE’s longer molecular chains. In designing this research, we aimed to improve the recovery of PSA film by introducing AE, which finally gains a significant elastic component of the viscoelastic behavior associated with the PSA/AE blends.

### 3.2. Non-Crosslinked Acrylic PSA/AE Blends

To establish the optimum content of AE, AE was mixed with acrylic PSA prepolymer without performing the crosslinking reaction. Table 2 contains the PSA/AE blended formulations. The samples were spread on PET films dried at 80 °C for 10 min and investigated in the absence of thermal crosslinking. This was done because AE, which has a relatively high molecular weight, affects adhesion performance and Young’s modulus of acrylic PSA.

**Figure 2:**
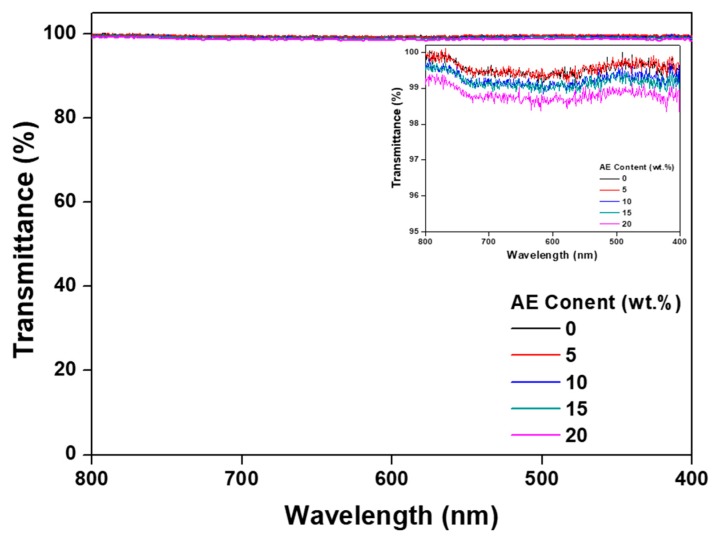
Transmittance in the visible range of non-crosslinked acrylic PSA/AE blends as a function of AE content.

Transparency of PSA is a highly important factor in display applications. Figure 2 shows the measured transmittance of the acrylic PSA/AE blends according to AE content. Transmittance measurements were taken after first establishing a baseline measurement using a pure PET film with no acrylic PSA coating. All the mixed samples, as clear films with no hazy aspect due to a very good mutual miscibility of the macromolecular components [17], exhibited transmittance values close to 100%, irrespective of AE content, which recommends them to be used as a PSA for display designing.

**Figure 3:**
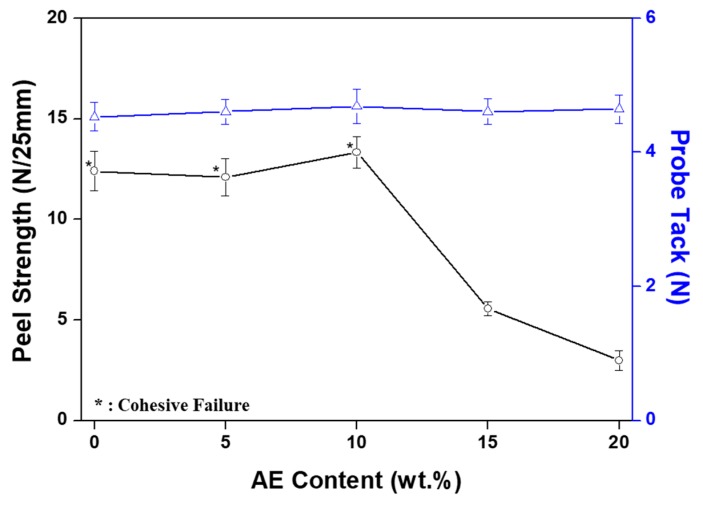
Peel strength and probe tack of non-crosslinked acrylic PSA/AE blends as a function of AE content.

Figure 3 shows the peel strength and probe tack results of acrylic PSA according to AE content. In the case of peel strength, cohesive failure occurred, although the adhesion property was maintained above 10 N/25 mm when AE content ranged from 0 to 10 wt %. Cohesive failure meant that the final product did not ensure reliability for application. This lack of cohesion was due to a dearth of entanglement because the acrylic PSA was not thermally processed in order to crosslink acrylic PSA prepolymer and the AE content was low. When the AE content was 15 wt %, the peel strength of acrylic PSA began to decrease and continuously decreased as AE content increased. This result is due to the aforementioned wettability of acrylic PSA being lowered by AE having a relatively large molecular weight. In the case of probe tack, there was no significant difference depending on the AE content, which is equivalent to saying that the long chains of the elastomer did not affect the cohesion properties up to 20 wt % of AE content. Such a behavior could be explained by a high miscibility of the blended components, with a very good inclusion of AE irrespective of its content, which practically did not affect the overall tackiness.

**Figure 4:**
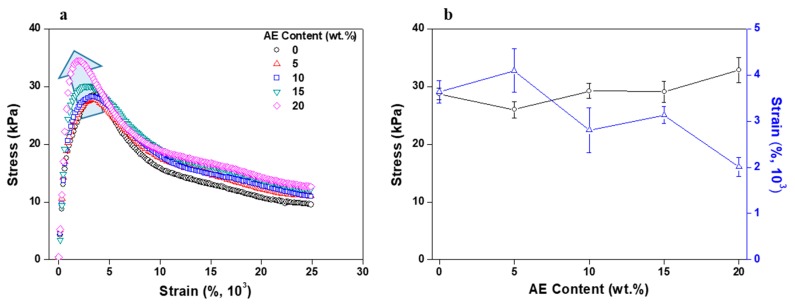
Lap shear test results for non-crosslinked acrylic PSA/AE blends as a function of AE content: (**a**) stress–strain curve and (**b**) maximum stress and strain values obtained at the maximum lap shear stress.

Figure 4 shows the results of the lap shear experiment on acrylic PSA according to the AE content. The shear stress increased continuously as the AE content increased, but its value was not large. This is because the acrylic PSA/AE blend is not thermally crosslinked. However, the shear stress increased with the inclusion of AE due to its long molecular chains associated with a certain degree of entanglement. In addition, the modulus of the acrylic PSA/AE blend increased slightly and caused the maximum shear strain to slightly decrease. Taking into account the effect of long polymer chains of AE on the adhesion properties of the mixed/non-crosslinked PSA/AE systems, we chose the compositions with 10 wt % AE as optimum ones on which the thermal crosslinking was performed.

**Figure 5:**
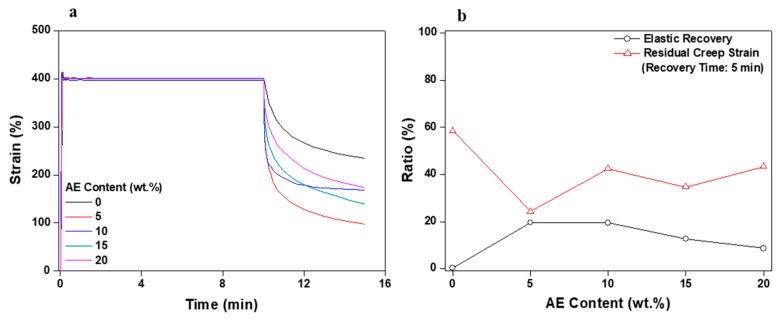
Stress relaxation test results for non-crosslinked acrylic PSA/AE blends as a function of AE content: (**a**) strain change and (**b**) elastic recovery and residual creep strain.

**Figure 6:**
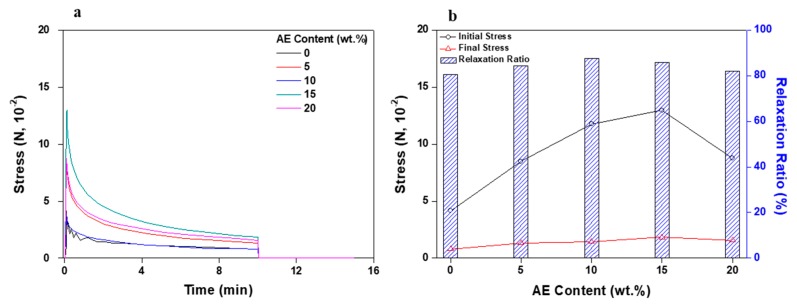
Stress relaxation test results for non-crosslinked acrylic PSA/AE blends as a function of AE content: (**a**) stress change and (**b**) initial stress, final stress, and relaxation ratio.

Figure 5 and Figure 6 display the results of recovery and stress relaxation measurements as a function of AE content. The data plotted in Figure 5a involving recovery measurements were acquired after maintaining the applied strain at a specific value (400%) for 10 min. Pure acrylic PSA prepolymer showed approximately 40% viscoelastic recovery as a result of residual creep strain. Compared with our previous results [14,15], this value is high considering it is pure acrylic PSA. This result is understandable because the acrylic PSA (received from the company specified earlier) is versatile and has basic adhesion and cohesive strength. At 5 wt % AE, the acrylic PSA/AE blend increased recovery for 5 min after removing the strain to approximately 80%, but as the AE content increased, the resilience decreased. The last aspect indicates that although the elasticity of the material itself should have improved by increasing the AE content, the disentanglement of AE chains occurred more easily at higher fractions of elastomer, most likely due to the weak attractive interactions between the AE molecules, having a direct consequence in a progressive decrease of the elastic recovery.

Figure 6 displays the results measuring the change in stress generated when a specific strain was applied for 10 min. As mentioned above, the modulus of the acrylic PSA/AE blend increased as the AE content increased, and the initial stress generated during specific strain continuously increased. Somehow unexpectedly, when the AE content increased to 20 wt %, the initial stress decreased. This peculiarity could be caused, as explained earlier, by an easier disentanglement undergone by the AE chains at high AE content.

### 3.3. Thermally Crosslinked Acrylic PSA/AE Blends

To improve the recovery of the acrylic PSA/AE blend, based on the results discussed above, the AE content was fixed at a certain value, and the acrylic PSA prepolymer was crosslinked. The AE content was fixed at 10 wt %, accounting for the adhesion performance and recovery results of the acrylic PSA/AE blend without crosslinking. Table 3 contains the crosslinked acrylic PSA/AE blends’ composition. By crosslinking acrylic PSA prepolymer, we attempted to investigate the adhesion properties and recovery behavior corresponding to the difference in bonding between the chains of the prepolymer and the AE.

**Figure 7:**
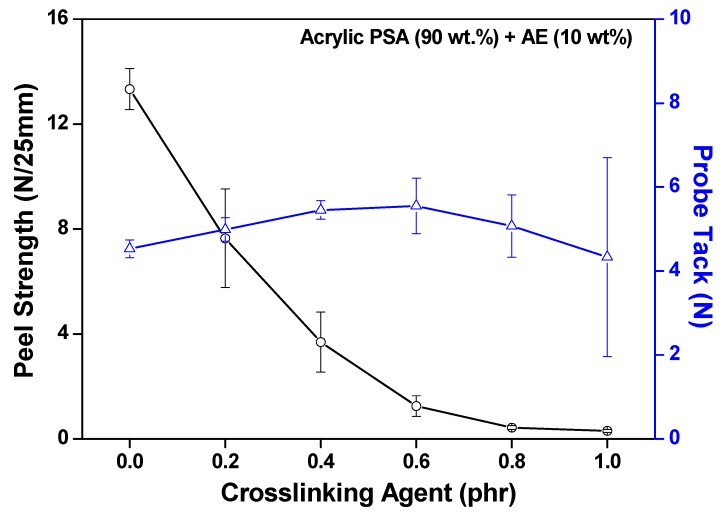
Peel strength and probe tack of acrylic PSA/AE blends as a function of crosslinking agent content.

Figure 7 shows the peel strength and probe tack measurement results of the acrylic PSA/AE blend dependent on the crosslinking agent content. Similar to typical crosslinked acrylic PSA, when 0.2 phr crosslinking agent was added, the peel strength began to decrease sharply. Thereafter, as the content of crosslinking agent increased, the peel strength also continued to significantly decrease. This result is due to a low wettability of the acrylic PSA prepolymer, which exhibits a weaker adhesion force as a consequence of the formation of a network during crosslinking. The probe tack tests showed a slight variation of the instantaneous adhesion, with a maximum in the range of 0.4–0.6 phr of crosslinking agent. The increase in the initial probe tack value was due to the crosslinking reaction increasing the cohesion of the short molecular chains of acrylic PSA. On the other hand, the reduction in probe tack value was caused by the excessive crosslinking which, in turn, led to a solidifying PSA/AE blend, similar to a typical polymer, and tackiness disappeared.

**Figure 8:**
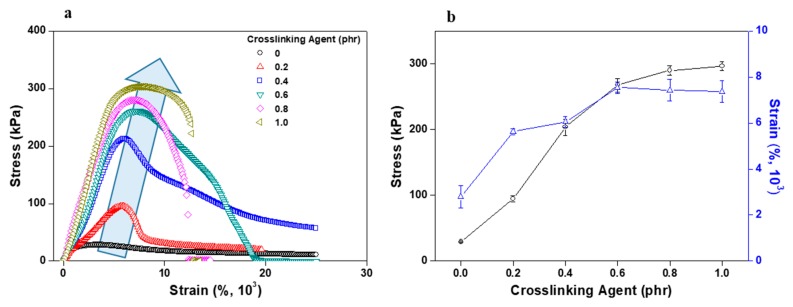
Lap shear test results for acrylic PSA/AE blends as a function of crosslinking agent content: (**a**) stress–strain curve and (**b**) maximum stress and strain values obtained at the maximum lap shear stress.

Figure 8 shows the results of the acrylic PSA/AE blend lap shear test as a function of crosslinking agent content. As the content of crosslinking agent increased, the shear stress and modulus value continued to rise. On the other hand, the strain at maximum stress began to continuously decrease, and when the content of the crosslinking agent was 0.8 phr or greater, a peeling phenomenon occurred, without stretching, after the maximum stress. This result, like the peel strength and probe tack results, was caused by the formation of the PSA prepolymer network with the crosslinking agent. This network plays a role similar to that of the AE molecular chains in the entangled state. However, the acrylic PSA/AE blend became rigid from excessive crosslinking when the content of the crosslinking agent was 0.8 phr or greater.

**Figure 9:**
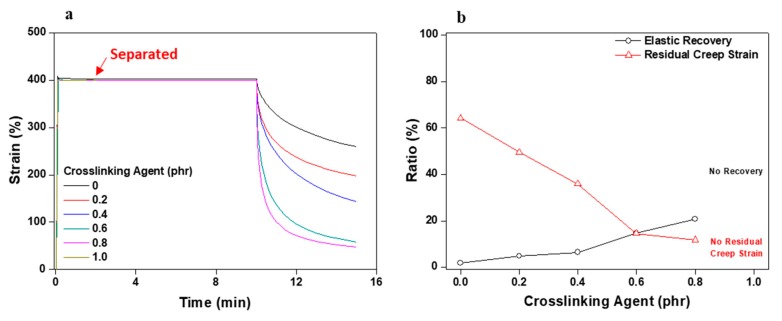
Stress relaxation test results for acrylic PSA/AE blends as a function of crosslinking agent content: (**a**) strain change and (**b**) elastic recovery and residual creep strain.

**Figure 10:**
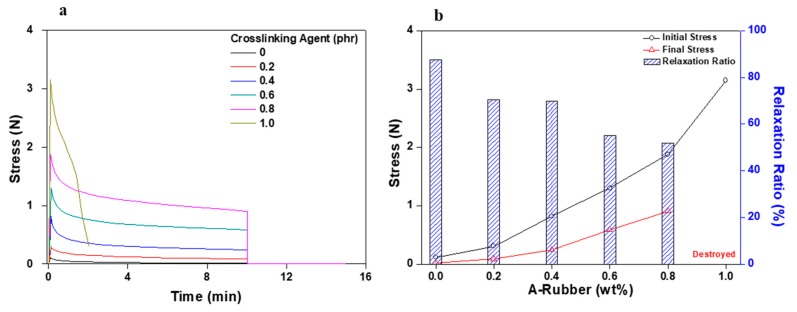
Stress relaxation test results for acrylic PSA/AE blends as a function of crosslinking agent content: (**a**) stress change and (**b**) initial stress, final stress, and relaxation ratio.

Figure 9 and Figure 10 graphically display the data of stress relaxation tests of the acrylic PSA/AE blend corresponding to the content of crosslinking agent. Overall, the results of these tests are significantly different from those for the acrylic PSA/AE blend without crosslinker. Figure 9 indicates the results measuring recovery when the constant strain is removed after its uninterrupted application for 10 min. Elastic recovery continuously improved as the content of the crosslinking agent increased. The residual creep strain also decreased drastically, with a maximum viscoelastic recovery of approximately 90% reached for the acrylic PSA/AE blend having 0.8 phr crosslinker. However, because of the too high rigidity attained at a crosslinker content of 1 phr, the acrylic PSA/AE system did not withstand at the applied strain (400%) and measuring both resilience and residual creep strain failed.

Figure 10 shows the changes in stress generated when a specific strain is applied for 10 min. Since the modulus of the acrylic PSA/AE blend increased as the content of crosslinking agent increased, the initial and final stresses also consistently increased. In addition, as expected, the relaxation ratio, which represents the elimination of initial stress, tended to decrease with increasing the content of the crosslinking agent. Eventually, the acrylic PSA/AE blend became very hard due to excessive crosslinking at a content of 1 phr crosslinking agent, and the initial stress value exceeded 3 N and shortly thereafter (approximately 2 min) the sample testing failed (see Figure 10a,b).

**Figure 11:**
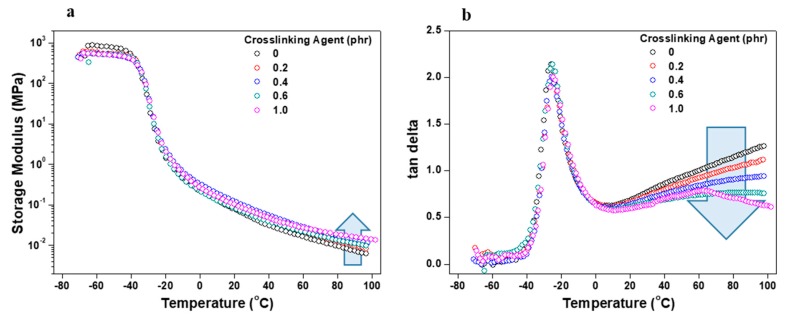
Viscoelasticity of acrylic PSA/AE blends on temperature at different contents of crosslinking agent: (**a**) storage modulus and (**b**) loss tangent values.

Figure 11 shows storage modulus and dissipation factor (tan delta) values according to the acrylic PSA/AE blend temperature at different crosslinking agent contents. In general, acrylic PSAs and AE are semisolid at room temperature and it is difficult to quantitatively evaluate their elasticity. Therefore, the viscoelasticity and glass-transition temperature of acrylic PSA/AE blends with crosslinking agent were analyzed through temperature sweep. As the content of crosslinking agent increased, the initial storage modulus did not differ greatly. However, as the crosslinking agent contents rose, the storage modulus increased continuously, and its change became larger in the high temperature region, as reported in a previous study [18]. On the other hand, the maximum value of loss tangent did not shift, whereas this quantity generally lowered as the content of the crosslinking agent increased in the high temperature region. This behavior is a direct consequence of an enhanced elasticity of the PSA/AE blend gained at high crosslinker contents, which eventually is also in accordance with a lower wettability of the same mixed adhesive. Based on this analysis, it is easy to understand why the test pieces with 1 phr crosslinking agent failed during the stress relaxation tests. These results also confirmed that the crosslinking agent controls elasticity and wettability, without affecting the glass-transition temperature of the acrylic PSA/AE.

## 4. Conclusions

To confirm the potential use of PSA in flexible displays, AE was applied to an acrylic PSA in order to assess adhesion performance and recovery. The transmittance in visible domain of the acrylic PSA/AE blend was approximately 100%, which demonstrates that the two components are mutually miscible, leading to a film completely devoid of any haze. Peel strength was maintained almost constant up to 10 wt % AE content, but cohesive failure then occurred and began to decline above 15 wt %. On the other hand, probe tack remained at the same value even when the AE content increased. The results show that the long chains of AE affected the penetration of acrylic PSA and that peel strength decreased, but the probe tack was maintained due to a phenomenon of entanglement. Reliable results for the stress relaxation test were difficult to obtain due to the weak bond between the molecular chains of acrylic PSA and AE. Based on the experimental results regarding the acrylic PSA/AE blend, the adhesion performance, recovery, and viscoelasticity were evaluated at a fixed AE content of 10 wt %, and crosslinking of the acrylic PSA prepolymer was examined as a function of crosslinking agent content. Thus, peel strength decreased continuously as the content of the crosslinking agent increased due to a decreased wettability. On the other hand, the probe tack displayed a small variation with crosslinker content, exhibiting maximum values within 0.4–0.6 phr of crosslinking agent. The lap shear test showed that shear stress and modulus increased constantly as the content of the crosslinking agent increased. Additionally, when the content of the crosslinking agent was 0.8 phr or greater, the PSA sheet became too rigid, and the test piece began to separate. The stress relaxation test indicated that elastic recovery and residual creep strain were continuously improved with the content of the crosslinking agent, and when a specific strain was set, the recovery enhanced up to approximately 90%. However, at a content of 1 phr crosslinking agent, the test piece did not withstand the specific strain and failed, even though the initial stress reached a maximum value of approximately 3 N. Viscoelasticity measurements revealed an ascending tendency for the storage modulus as the content of the crosslinking agent increased, with no effect on the glass-transition temperature of the mixed systems.

According to the results of this study, we confirmed that the development of an acrylic PSA for use in flexible displays is possible using an elastomer with a large molecular weight. In addition, the molecular weight of both the acrylic PSA and the elastomer, along with the crosslinking method, were used to prove that adjustment of the adhesion performance and recovery of the PSA is also feasible in designing flexible displays.

## Figures and Tables

**Table 1:** Characterization of the acrylic PSA prepolymer and AE.

Materials	*M_n_* (g/mol) ^*^	*M_w_* (g/mol) ^*^	PDI ^*^	*T_g_* (°C) ^**^
Acrylic PSA prepolymer	24,375	50,670	2.1	−40
Acrylic elastomer	342,750	1,167,800	3.4	−30

Molecular weights (M_n_ and M_w_) and polydispersity indices (PDIs) were measured by GPC ^*^; *T_g_* was measured by differential scanning calorimetry (DSC) ^**^.

**Table 2:** Composition of acrylic PSA/AE blends without crosslinking agent.

Samples	Acrylic PSA Prepolymer (wt %)	Acrylic Elastomer (wt %)
PSA-AE-00	100	0
PSA-AE-05	95	5
PSA-AE-10	90	10
PSA-AE-15	85	15
PSA-AE-20	80	20

**Table 3:** Composition of acrylic PSA/AE blends with crosslinking agent.

Samples	Crosslinked Acrylic PSAs		AE (wt %)
	Acrylic PSA Prepolymer (wt %)	Crosslinking Agent (phr)	
PSA-AE-10-00	90	0	10
PSA-AE-10-02		0.2	
PSA-AE-10-04		0.4	
PSA-AE-10-06		0.6	
PSA-AE-10-08		0.8	
PSA-AE-10-10		1.0	
